# Numerical Simulation on Thermal Curing Behavior and Defect Formation Mechanism Analysis of Basalt Fiber-Reinforced Polymer Core Rods for Composite Cross-Arms

**DOI:** 10.3390/polym18161953

**Published:** 2026-08-09

**Authors:** Mingjia Zhang, Yao Duan, Zengsheng Zhang, Dingwei Fu

**Affiliations:** State Grid Zhejiang Electric Power Co., Ltd. Construction Company, Citizen Street No. 219, Hangzhou 310000, China; 18868824306@163.com (Y.D.); powerzzs@126.com (Z.Z.); m13646751530@163.com (D.F.)

**Keywords:** basalt fiber, composite cross-arms, curing kinetics, defect formation mechanism, degree of cure, finite element method, pultrusion process, thermal simulation

## Abstract

The production of basalt fiber-reinforced polymer (BFRP) core rods for composite cross-arms in power transmission lines currently suffers from high defect rates—including internal porosity, surface cracking, under-curing, and thermal yellowing. Although BFRP exhibits superior mechanical strength, thermal stability, and corrosion resistance compared with conventional glass-fiber-reinforced polymers, industrial-scale manufacturing of BFRP core rods remains immature; critically, the spatial–temporal evolution of thermal curing behavior inside the mold has not been systematically characterized. This knowledge gap relies on costly, time-consuming trial-and-error process tuning. This study develops a validated numerical simulation method grounded in an autocatalytic curing kinetics model, calibrated using differential scanning calorimetry (DSC) experiments, to quantitatively predict the spatiotemporal distribution of temperature and degree of cure during pultrusion-based BFRP core rod fabrication. Four key curing metrics—final surface degree of cure, pre-cure degree, peak core temperature, and through-thickness cure gradient—are identified and rigorously evaluated against critical process parameters (pultrusion speed, pre-cure and cure zone temperatures, and axial temperature gradient) via an L_9_ orthogonal experimental design. The model elucidates four distinct defect formation mechanisms, thereby reducing empirical dependence and accelerating the reliable industrial deployment of BFRP core rods in composite cross-arm systems.

## 1. Introduction

The replacement of steel with fiber-reinforced composite materials (FRCMs) in the construction of transmission lines can effectively reduce carbon emissions generated during the construction phase, thereby holding significant implications for the development of an environmentally friendly power grid and the realization of dual-carbon targets [1,2]. Currently, the application of FRCMs in transmission lines is primarily concentrated in composite insulators, composite cross-arms, and composite poles. Among these, composite insulating cross-arms have gradually been adopted due to their superior insulation properties, as well as ease of installation and maintenance [1]. Multiple application studies have demonstrated that they can effectively reduce the lightning-induced tripping rate of distribution networks and enhance the reliability of power supply [3,4].

Composite insulating cross-arms are mainly composed of silicone rubber sheds, fiber-reinforced composite core rods, and metal fittings. The core rod is a critical component of the composite insulating cross-arm, as it not only provides the required structural strength but also contributes to the internal insulation strength of the cross-arm [5]. In the current market, core rods for composite insulating cross-arms are typically manufactured via the pultrusion of untwisted glass fiber roving impregnated with epoxy resin [6]. However, due to the inherent limitations in the mechanical properties of glass fibers, glass fiber core rods exhibit significant deflection under load [7]. This leads to excessive end displacement when composite insulating cross-arms are applied in corner towers, areas with high line loads such as mountainous regions with strong wind fields, or under natural disasters like typhoons. Additionally, studies have indicated that glass fibers are relatively weak in chemical corrosion resistance and high-temperature performance, which results in multi-stress corrosion cracking in acidic and humid-heat environments [8,9]. The substitution of glass fibers with basalt fibers can effectively address the aforementioned issues. Basalt fibers are produced by melting natural volcanic rock at a high temperature of 1500 °C and then drawing the molten material into fibers [10]. As a green strategic resource, basalt fibers have been widely utilized in industries such as aircraft manufacturing, radar production, and filtration/adsorption materials [11]. Their environmental friendliness, excellent mechanical properties, thermal stability, and acid resistance make them a promising alternative to glass fibers [12].

Previous research has extensively investigated the application of epoxy resin-based basalt fiber-reinforced composites (BFRPs) in core rods. Some research compared the mechanical and durability properties of basalt fiber and glass fiber core rod materials with the same areal density, confirming the advantages of basalt fibers over glass fibers [13,14]. There is research which prepared basalt fiber-reinforced modified polyurethane-based composite insulator core rods and demonstrated their effectiveness in large-diameter core rod applications [15]. Some researchers optimized the resin formulation for basalt fiber core rods, which effectively improved the service life of basalt fiber cross-arms in coastal areas [16]. By designing the structure of basalt fiber, composite cross-arms could enhance their tolerance under typhoon conditions [17,18].

Beyond experimental material characterization, numerical simulation has emerged as a powerful tool for understanding and optimizing the pultrusion process of fiber-reinforced polymer composites. Finite element modeling has been extensively applied to predict the mechanical performance of pultruded profiles, including the shear behavior, the transverse tensile properties, and the lateral torsional buckling behavior [19,20,21,22]. These studies have demonstrated the capability of numerical methods to capture complex structural responses and to supplement experimental investigations. In the context of performance optimization, finite element simulations have also been employed to evaluate the application of pultruded composites in large-scale wind turbine blades, enabling weight reduction and structural efficiency improvements [23]. Regarding the curing process itself, recent efforts have focused on the numerical simulation of cure kinetics and curing deformation. Izadi et al. [24] conducted experimental and numerical investigations on the cure kinetics of thermoplastic fiber-reinforced composites during pultrusion, establishing kinetic models for Elium^®^-based systems. Liu et al. [25] combined design, simulation, and experiments to optimize the tube-making pultrusion process with glass fabric/polypropylene composites. Wu et al. [26,27] developed thermal–chemical–structural coupled finite element models to simulate curing deformation in curved composite plates and polyurethane composite solar cell bezels, respectively, employing orthogonal experimental designs to identify optimal process parameters for minimizing residual stress and deformation. Despite these advances, the vast majority of existing simulation studies have focused on glass fiber-reinforced or carbon fiber-reinforced systems, with limited attention paid to basalt fiber-reinforced polymer (BFRP) composites. Furthermore, while these studies have addressed various aspects of mechanical performance and curing deformation, a systematic numerical framework that correlates the spatiotemporal evolution of temperature and curing degree with the formation mechanisms of multiple defect types—particularly internal porosity, surface cracking, under-curing, and thermal yellowing—in BFRP core rods has not yet been established.

Although basalt fiber-reinforced polymer (BFRP) offers compelling advantages for composite cross-arm applications, industrial-scale manufacturing of BFRP core rods remains technologically immature. The core scientific challenge stems from the absence of systematic, quantitative characterization of the spatiotemporal evolution of temperature and degree of cure during pultrusion—a gap that directly undermines predictive process control. Consequently, manufacturers are compelled to rely on costly, iterative trial-and-error parameter tuning, resulting in batch-to-batch quality variability and persistently high defect rates (e.g., internal porosity, surface cracking, and under-curing). The overarching practical challenge, therefore, is the lack of a rational, mechanism-based process-design framework capable of guiding both production optimization and root-cause-driven defect mitigation in BFRP core rod fabrication.

To bridge this science–practice gap, this study establishes and experimentally validates a physics-informed numerical simulation framework grounded in autocatalytic curing kinetics. The model quantitatively predicts the coupled thermal–chemical evolution within the die and along the rod length under industrially relevant pultrusion conditions. By enabling mechanistic interpretation of process–structure–property relationships, this framework provides a transferable methodology for predictive parameter optimization—reducing empirical dependence, enhancing reproducibility, and accelerating the robust industrial deployment of BFRP core rods in high-reliability composite cross-arm systems.

## 2. Materials and Methods

### 2.1. Core Rod Preparation

#### 2.1.1. Materials

Basalt fiber roving: specification of 9600 tex, single-filament diameter of 13 μm, density of 2.8 g/cm^3^, with epoxy silane sizing agent (Zhejiang Perlim Electric Technology Co., Ltd., Quzhou, China); glass fiber roving: specification of 9600 tex, single-filament diameter of 13 μm, density of 2.54 g/cm^3^, with epoxy silane sizing agent (Zhejiang Perlim Electric Technology Co., Ltd., Quzhou, China); bisphenol A epoxy resin (DGEBA): epoxy value of 0.51 mol/100 g, supplied by Zhejiang Perlim Electric Technology Co., Ltd.; curing agent: methylhexahydrophthalic anhydride (MHHPA), supplied by Zhejiang Perlim Electric Technology Co., Ltd.; accelerator: 2,4,6-tris(dimethylaminomethyl)phenol (DMP-30), supplied by Zhejiang Perlim Electric Technology Co., Ltd.; internal mold release agent: polydimethylsiloxane (PDMS) (Zhejiang Perlim Electric Technology Co., Ltd., Quzhou, China).

#### 2.1.2. Preparation Process

**Figure 1:**
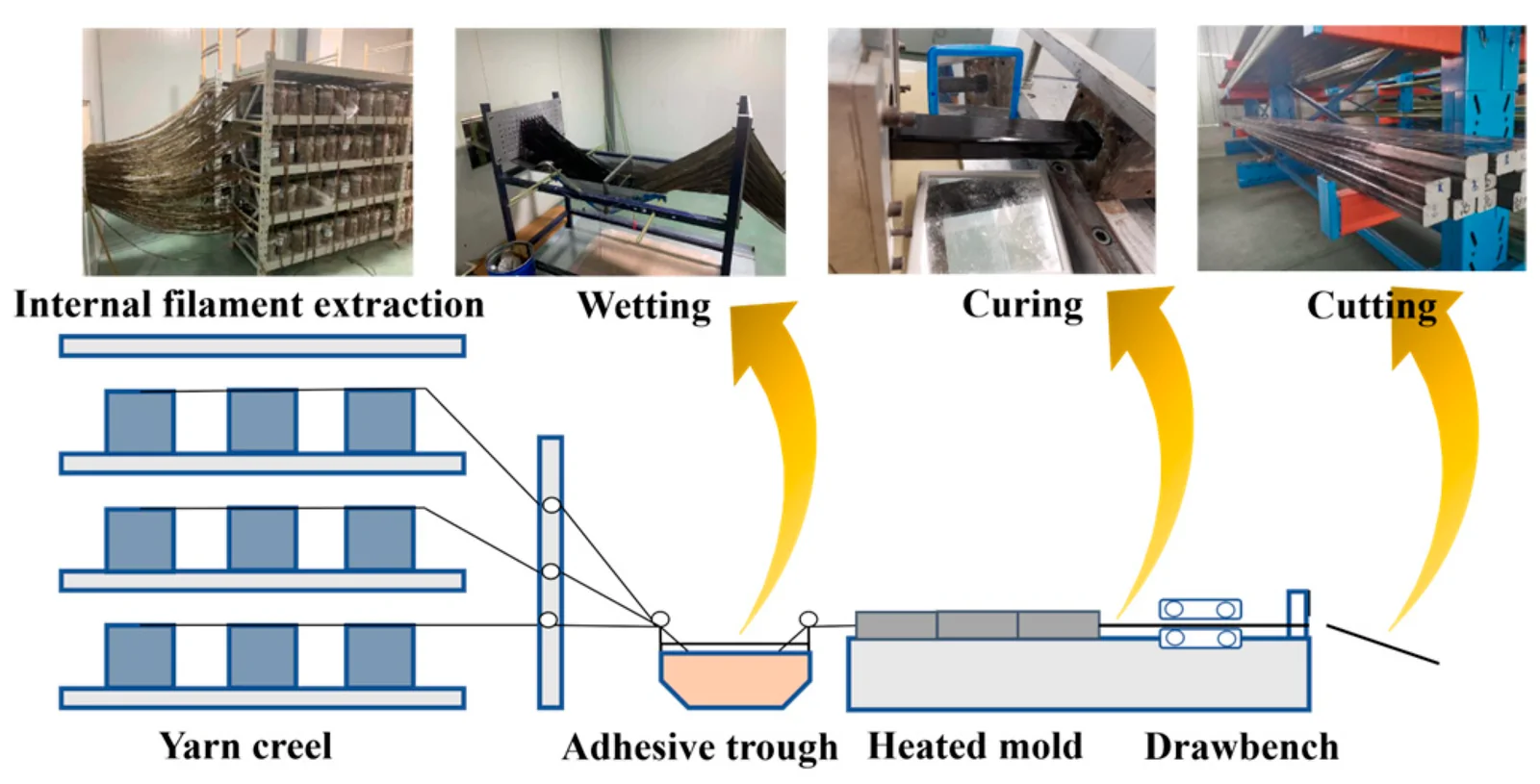
Schematic diagram of the pultrusion production process.

Core rod samples were fabricated via the pultrusion process, and the pultrusion production line is illustrated in Figure 1. The matrix resin mixture was prepared with a mass ratio of DGEBA:MHHPA:DMP-30:PDMS = 55:40:3:1. The formulated resin mixture was poured into the resin tank. Basalt fiber roving was mounted on the creel, arranged through a yarn-threading plate, and then introduced into the resin tank for impregnation. The impregnated roving was cured in a mold to form continuous pultruded rods, which were then pulled by a traction machine (Zhejiang Perlim Electric Technology Co., Ltd., Quzhou, China) to enable continuous sample preparation. During the fabrication process, the environmental humidity around the creel and resin tank was maintained below 40% to avoid moisture absorption by the roving and resin mixture, which could impair the performance of the final product. A three-section heating mold was employed.

#### 2.1.3. Common Defects in the Fabrication of Basalt Fiber Core Rods

**Figure 2:**
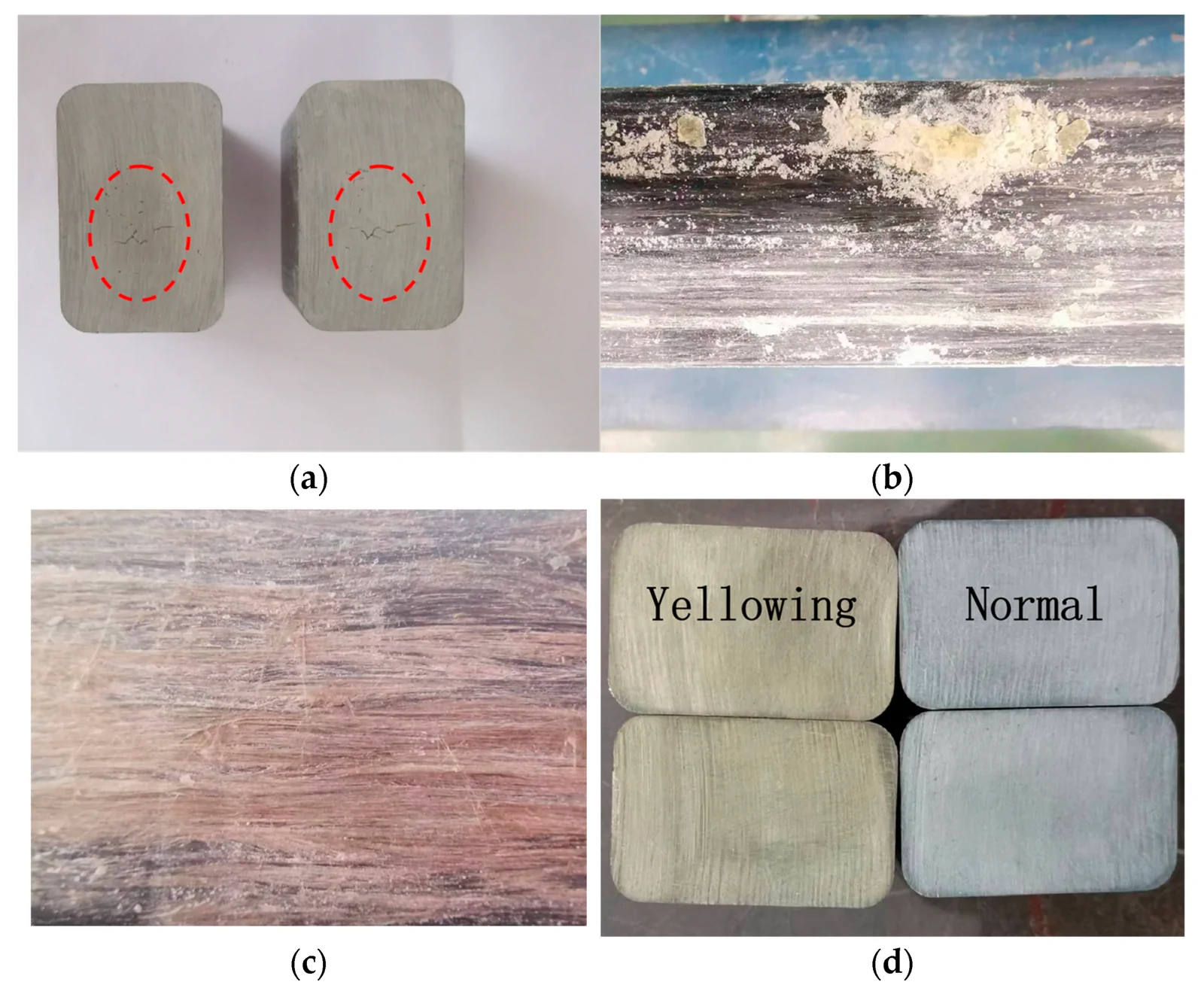
Defects in pultrusion molding of composite core rods: (**a**) Internal crack; (**b**) surface crack; (**c**) surface fiber exposure; (**d**) yellowing.

Given the absence of industrialized manufacturing of basalt fiber core rods, there remains a lack of a rational model to characterize the core rod forming process inside the mold during the production of basalt fiber-based power composite core rods. This results in a certain degree of subjectivity in the control of process quality during the current production of cross-arm core rods. Due to the limited experience in basalt fiber core rod production, the final forming temperature and curing degree of the product cannot be regulated based on prior production experience, leading to the occurrence of defects. As illustrated in Figure 2, multiple types of defects persist during production, including internal cracks (Figure 2a), surface cracks (Figure 2b), surface fiber exposure (Figure 2c), and yellowing (Figure 2d).

The resolution of the above four types of defects is typically achieved through empirical judgment and trial-and-error adjustments of production process parameters. However, due to the scarcity of production experience with basalt fiber core rods and the significant differences in their thermal properties compared to glass fibers, adjusting the production process leads to issues such as excessive cycle time and cost investment. The use of numerical simulation methods for the optimal design of process parameters enables quantitative control over the temperature and curing degree during product forming, thereby avoiding the formation of the aforementioned defects.

### 2.2. Experiment

#### 2.2.1. Thermal Performance Testing

This study analyzed the heat release characteristics of the resin and obtained the residual curing degree of the core rod through DSC tests. The detailed procedure is as follows:

The curing exothermic process of the resin mixture was measured using the Netzsch DSC3500 differential scanning calorimeter (NETZSCH, Selb, Germany). The protective gas was nitrogen with a flow rate of 10 mL/min. The temperature range was from 25 °C to 250 °C, and the heating rates were 5 K/min, 10 K/min, 15 K/min, and 20 K/min. The total reaction heat *H* can be obtained by calculating the average value of the heat released.

The determination of the degree of curing requires taking approximately 20 milligrams of samples from both the center and the edge of the core rod and then conducting a DSC test. The average value of the heat released during the process is calculated to obtain the remaining heat *H_r_*. The actual curing degree can be calculated using Equation (1).

(1) $$\alpha =\frac{\left(1-V\right)H-H_{r}}{\left(1-V\right)H}$$

#### 2.2.2. Mechanical Performance Testing

To investigate the mechanical differences between defective and intact core rods, bending tests were conducted in accordance with standard Q/GDW12069-2020 [28] to determine the ultimate bending stress at fracture. Specimens were mounted on a bending–torsion testing machine, with the load applied perpendicular to the longitudinal axis of the rods. The load was increased steadily until fracture, and the maximum stress was recorded.

#### 2.2.3. Insulation Performance Testing

To compare the insulation performance of defective and normal core rods, a water diffusion test was carried out following IEC 62217-2012 [29]. The core rods were cut into 30 cm long specimens, which were then immersed in a 0.1 wt% NaCl aqueous solution and kept boiling for 100 h. An AC voltage of 12 kV was applied to the specimens both before and after the diffusion test, and the leakage current amplitude was recorded using a digital multimeter (RIGIO DM3068) (RIGOL TECHNOLOGIES CO., LTD., Suzhou, China). A leakage current below 100 μA was considered to meet the acceptance criterion.

### 2.3. Numerical Simulation of Thermal-Curing Degree for Basalt Fiber Core Rods

#### 2.3.1. Model Establishment

**Figure 3:**
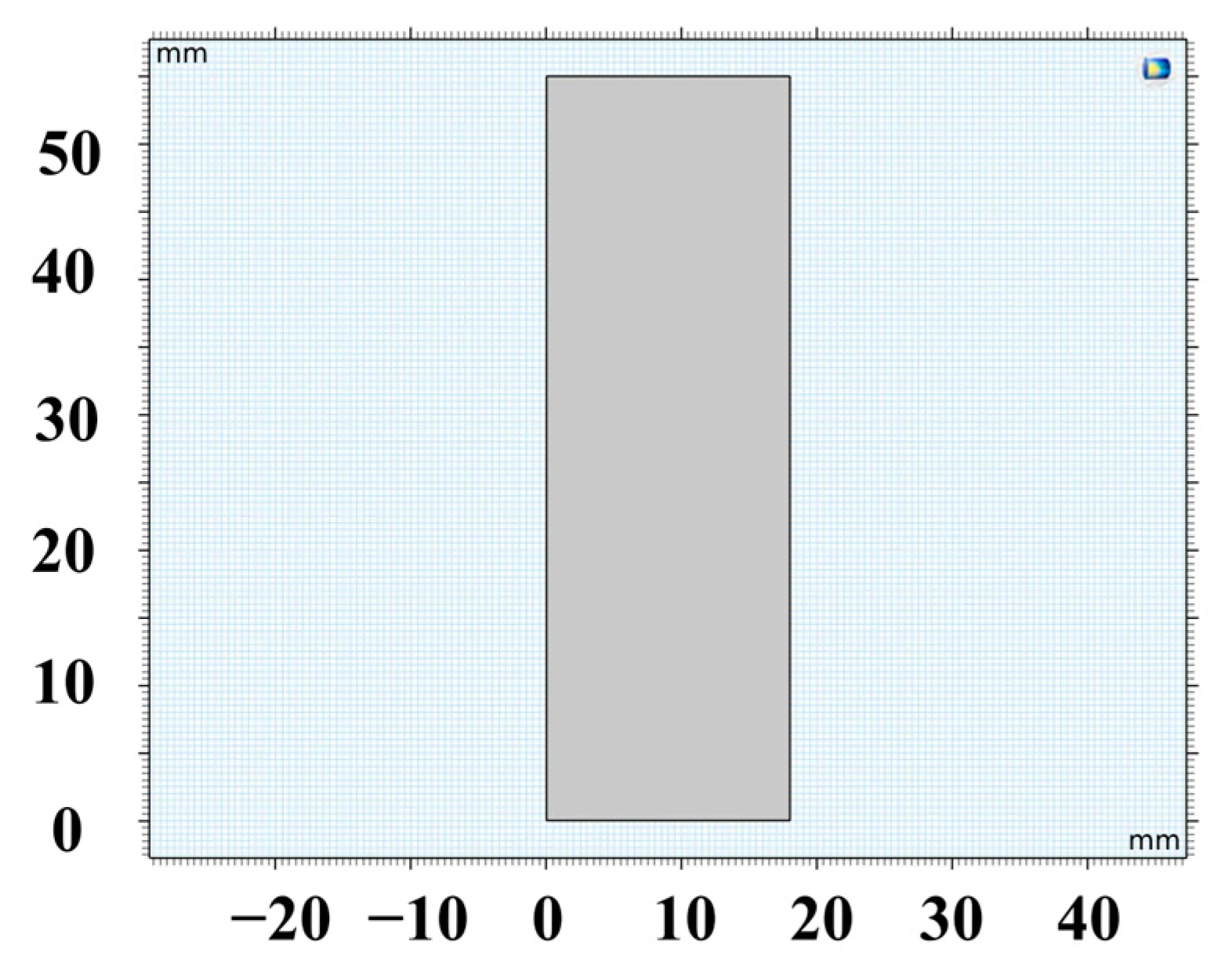
Two-dimensional axisymmetric model.

The finite element analysis of the thermal-curing degree field during core rod curing was performed using Comsol Multiphysics^®^ v6.4. The pultrusion process in actual production was simplified for analysis as follows: (1) Although the resin undergoes viscosity changes due to phase transition, the traction machine used in the current process operates at a constant speed, so the variation in traction speed caused by resin phase transition is neglected. (2) Due to heat transfer with air, the temperature environment of the fibers when pulled near the mold entrance is no longer at room temperature. However, since the external temperature does not exceed 100 °C, the curing reaction remains in the endothermic stage, and its impact on the curing degree is negligible. Therefore, only the curing and forming process of the core rod inside the mold is simulated. (3) As the pultrusion process of the core rod is a constant-cross-section forming process, the curing and forming process of a single cross-section can represent that of the entire composite core rod inside the mold. Thus, as shown in Figure 3, the core rod curing model can be reduced to a two-dimensional axisymmetric model.

#### 2.3.2. Internal Heat Source Equation for Core Rod Curing

Models for characterizing the resin curing reaction can be broadly classified into two categories: mechanism and phenomenology. Mechanistic models are derived from the heat absorption and release associated with chemical bond changes; they provide high accuracy in studying reaction pathways but tend to yield significant errors in predicting macroscopic heat release and are difficult to integrate into macroscopic physical field calculations. Phenomenological models, in contrast, are established on the basis of macroscopic thermal behavior and disregard the underlying reaction chemistry. Although such models lack clear physical interpretability, they exhibit strong versatility when applied to macroscopic physical field simulations.

In this study, a phenomenological model is employed to establish the exothermic equation for core rod curing. Given that the curing rate of the core rod is nearly zero at the initial stage of the reaction, an autocatalytic curing reaction model [30] is selected, as shown in Equation (2), to characterize the internal heat source during core rod curing.

(2) $$\frac{d\alpha }{dt}=\frac{dH}{H_{\mathrm{all}}dt}=Ae^{\left(\frac{-E}{RT}\right)}\alpha^{m}{\left(1-\alpha \right)}^{n}$$

In this equation, *A*, *m*, and *n* are model parameters, where *A* is the reaction rate factor, and *m* and *n* are the reaction orders; *E* is the activation energy; *R* is the ideal gas constant; *T* is the absolute temperature; *α* is the degree of reaction, i.e., the cure degree; *t* is the reaction time; *H* is the heat released; and *H_all_* is the total reaction heat.

**Figure 4:**
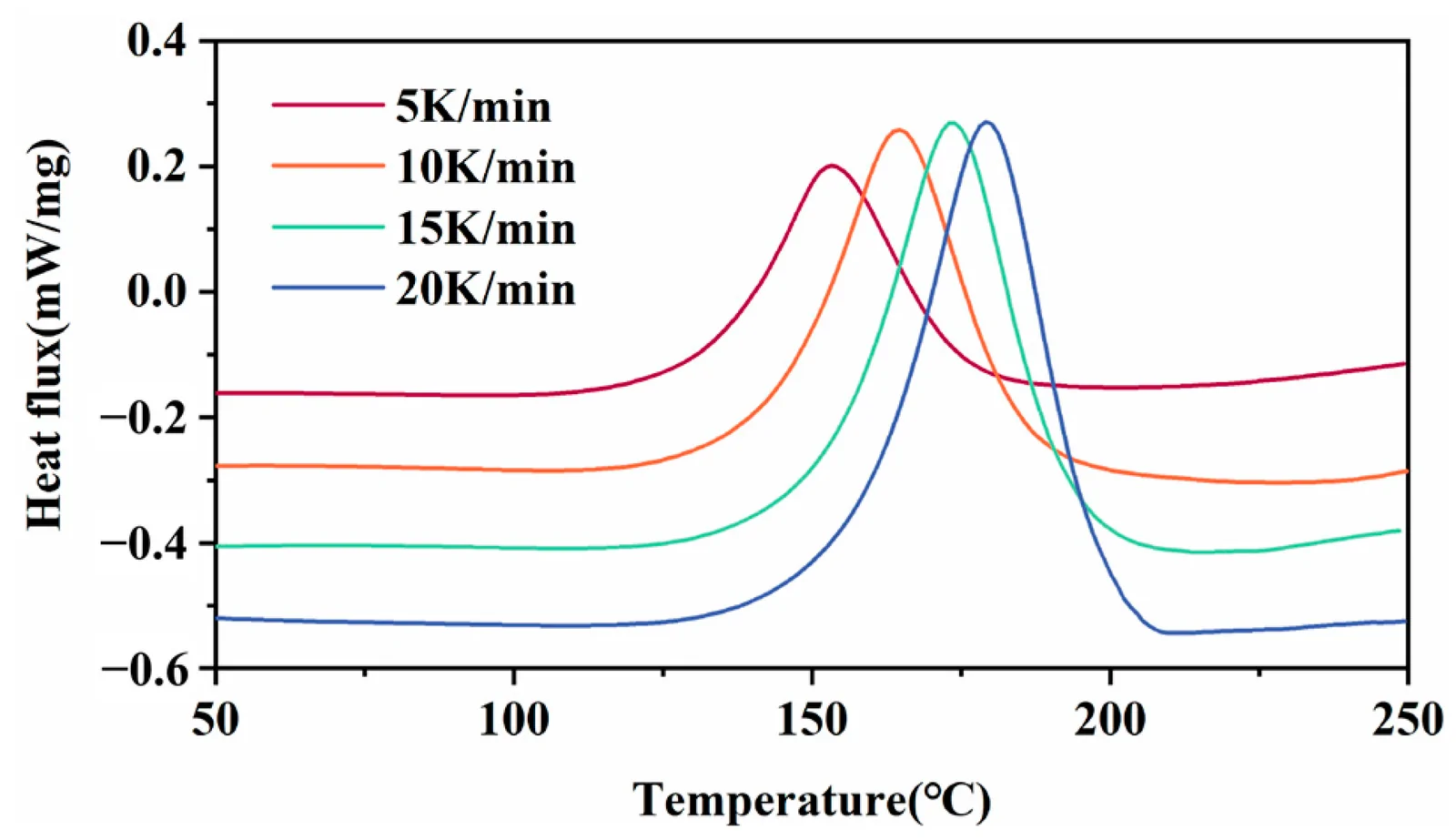
DSC curves at different heating rates.

The heat release curve of the core rod solidification is shown in Figure 4. The characteristic parameters of the core rod’s curing exothermic behavior obtained from the DSC tests are shown in Table 1. The average value of the measured total reaction heat Hall was determined to be 112.205 J/g.

The reaction activation energy E was obtained using the Kissinger method [31]. Taking the logarithm of Equation (3) yields Equation (4). Taking the logarithm of Equation (2) gives Equation (5).

(3) $$\begin{array}{c} \frac{E\beta }{RT_{t}^{2}}=Ae^{\frac{-E}{RT}}\\ \frac{E\beta }{RT_{t}}=Ae^{\frac{-E}{RT}} \end{array}$$

(4) $$ln\left(\frac{\beta }{T_{t}^{2}}\right)=ln\left(\frac{AR}{E}\right)-\frac{E}{RT_{t}}$$

(5) $$ln\left(\frac{d\alpha }{dt}\right)+\frac{E}{RT}=ln\left(A\right)+mln\left(\alpha \right)+nln\left(1-\alpha \right)$$

In the equation, *β* is the heating rate and *T_t_* is the peak temperature.

**Figure 5:**
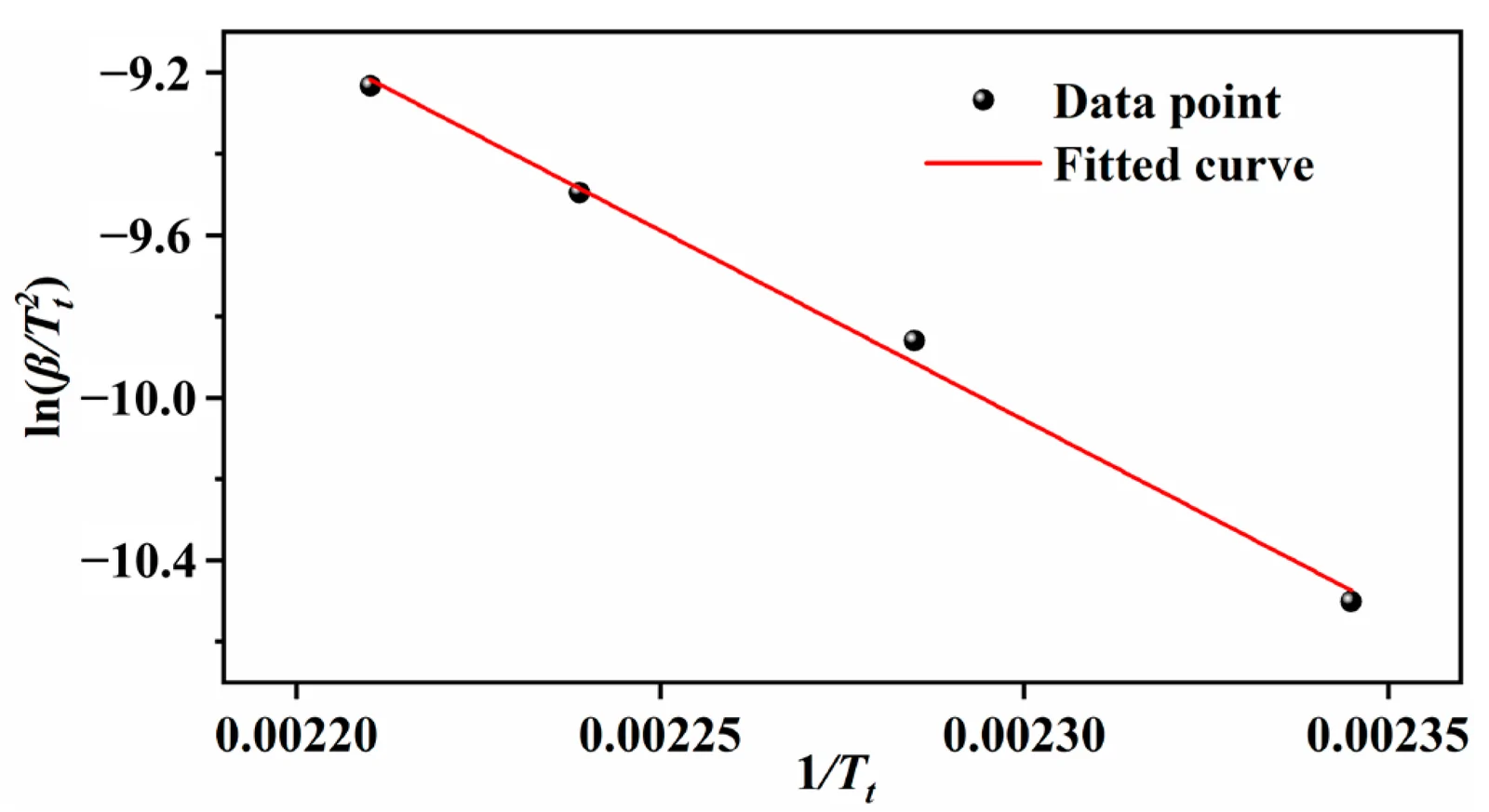
Fitted curve.

As shown in Figure 5, when taking $ln\left(\beta /T_{t}^{2}\right)$ as the dependent variable and $1/T_{t}$ as the independent variable, the linear fit exhibits a high coefficient of determination (R^2^ = 0.904), the fitted curve −E/R as a slope of −9321, giving a reaction activation energy E of 77.499 kJ/mol.

Based on Equation (4), curve fitting was conducted with $ln\left(d\alpha /dt\right)+E/\left(RT\right)$ as the dependent variable and *α* as the independent variable. The resulting fitting parameters *A*, *m*, and *n* are presented in Table 2.

After averaging the parameters, the internal heat source equation is derived as shown in Equation (6).

(6) $$\frac{d\alpha }{dt}=3.05\times 10^{7}e^{\frac{-77499}{8.314T}}\cdot \alpha^{0.388}\cdot {\left(1-\alpha \right)}^{0.964}$$

#### 2.3.3. Equations for Thermal-Curing Degree Field Quantities and Boundary Conditions

Given that the boundary temperature and internal heat source during the core rod curing process involve time-dependent temperature variations, the fundamental equation for the two-dimensional temperature field of the core rod is a transient heat transfer equation. Based on the internal heat balance derived from the first law of thermodynamics, and with the internal heat source being inversely proportional to the fiber content, the governing equation is presented in Equation (7).

(7) $$\rho C\frac{\partial T}{\partial t}=\lambda \left(\frac{\partial^{2}T}{\partial x^{2}}+\frac{\partial^{2}T}{\partial y^{2}}\right)+(1-V)\frac{\partial Q}{\partial t}$$

In the equation, *ρ* is density, *C* is specific heat capacity, *T* is temperature in Kelvin, *V* is the volume fraction of fibers, *Q* is the internal heat source, and *t* is time. Substituting Equation (5) into Equation (6) gives rise to Equation (8).

(8) $$\frac{\partial Q}{\partial t}=\rho \frac{dH\left(t\right)}{dt}=\rho H_{all}\frac{d\alpha }{dt}=\rho H_{all}Ae^{\left(\frac{-E}{RT}\right)}\alpha^{m}{\left(1-\alpha \right)}^{n}$$

The pultrusion process is carried out in a controlled environment with constant temperature and humidity. During normal production, the change in environmental temperature has a negligible impact on the formation of the core rod. Moreover, the temperature field of the core rod is mainly determined by heat transfer between the heating mold wall and the surface of the core rod, as well as the internal heat transfer of the core rod. Therefore, in this model, radiation’s consequences are also ignored.

During the core rod solidification process, the fibers are in close contact with the mold wall, and the un-solidified resin also has sufficient fluidity to fill any tiny surface irregularities, thereby reducing the effective contact resistance. Therefore, in this paper, the interface between the mold and the core rod is regarded as the ideal thermal contact surface.

Based on the above considerations, the temperature of the model’s outer surface is known, satisfying the first-kind boundary condition for the thermal field. During the curing process, process parameters including mold temperature and drawing speed are characterized by the temperature variation function of the outer side. The boundary conditions for the thermal field are presented in Equation (9).

(9) $$T\left(x,y,t\right)=T(v,s)$$

In the equation, *t* is time; *v* is the pulling speed; and *s* is the mold length.

At the initial time, the temperature inside a specific cross-section equals the ambient temperature, and the degree of cure corresponds to the initial degree of cure, as expressed in Equations (10) and (11).

(10) $$T\left(x,y,0\right)=T_{0}$$

(11) $$\alpha \left(x,y,0\right)=0.01$$

#### 2.3.4. Mesh Division and Material Parameters

**Figure 6:**
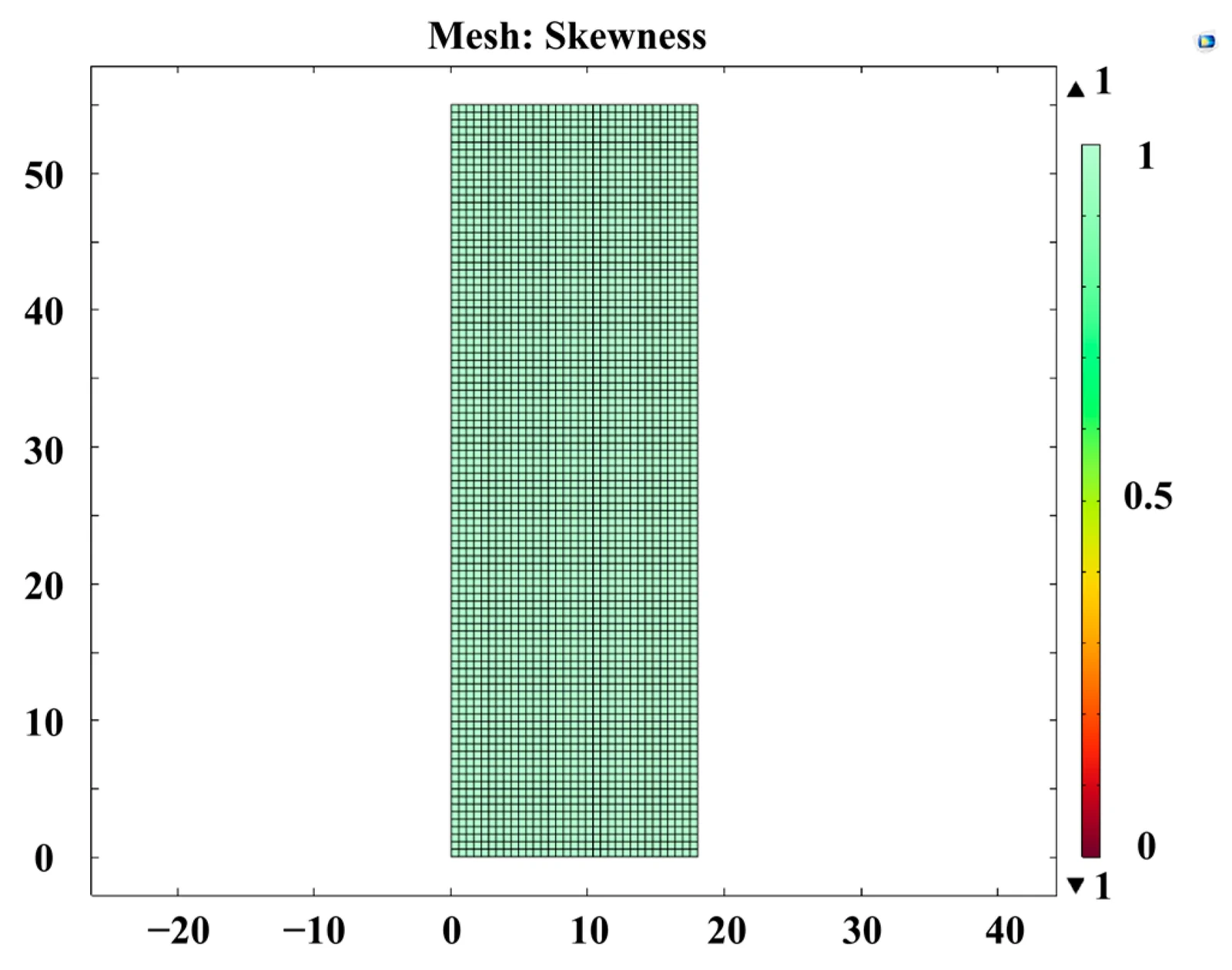
Mesh.

A quadrilateral mapped mesh was adopted for the 2D axisymmetric domain. The mesh consists of 3300 quadrilateral elements with 3434 vertices. The mesh quality was evaluated using the skewness metric, with the distribution presented in Figure 6. The skewness values are concentrated below 0.3, indicating good mesh quality and sufficient resolution for the transient thermal simulation. The typical computational time for a single simulation case is approximately 60 s on a standard workstation (Intel Core i7-10700, 32 GB RAM).

During the curing process, the fiber-reinforced resin gradually transitions into a composite, altering its internal thermal properties. In simulation calculations, however, assigning distinct thermal parameters to the fibers and resin individually is not feasible. Consequently, when defining the material properties, all performance parameters within the mold are assumed to remain constant and are determined based on a homogenized averaging effect. The density and specific heat capacity of the material inside the mold are calculated using Equation (12), while its thermal conductivity is taken to be identical to that of the resin.

(12) $$\varphi =\varphi_{fiber}V+\varphi_{resin}\left(1-V\right)$$

In the equation, φ is density or specific heat capacity, $\varphi_{fiber}\;$ is the corresponding properties of the fibers, $\varphi_{resin}\;$ is the corresponding properties of the resin, and *V* is the volume content of the fibers. The required material parameters are shown in Table 3.

## 3. Results

### 3.1. Model Accuracy Analysis

Given that real-time monitoring of the curing degree inside the mold is impractical during the production process, the curing degree was instead determined experimentally using specimens prepared under specific production conditions. The measured values were subsequently compared with the simulated predictions to validate the accuracy of the established kinetic model. Employing the process parameters listed in Table 4, core rod samples were fabricated, and the relative errors between the predicted and experimentally measured curing degrees were calculated.

The discrepancies between simulated and experimentally measured curing degrees arise from several simplifications. Thermophysical properties (density, specific heat, thermal conductivity) were treated as constants rather than temperature- or cure-dependent, potentially introducing systematic deviations, especially during the later exothermic stage. The 2D axisymmetric model neglects axial heat conduction between cross-sections, an effect most significant at steep temperature gradients between heating zones. The omission of resin volumetric shrinkage may also subtly affect thermal boundary conditions. Despite these factors, the overall error remains within 10%, confirming the model’s adequacy for predicting curing characteristics and defect mechanisms. This level of accuracy is considered acceptable, indicating that the current model is sufficiently reliable for further process analysis and optimization.

### 3.2. Analysis of Key Characteristic Quantities in the Curing Process

**Figure 7:**
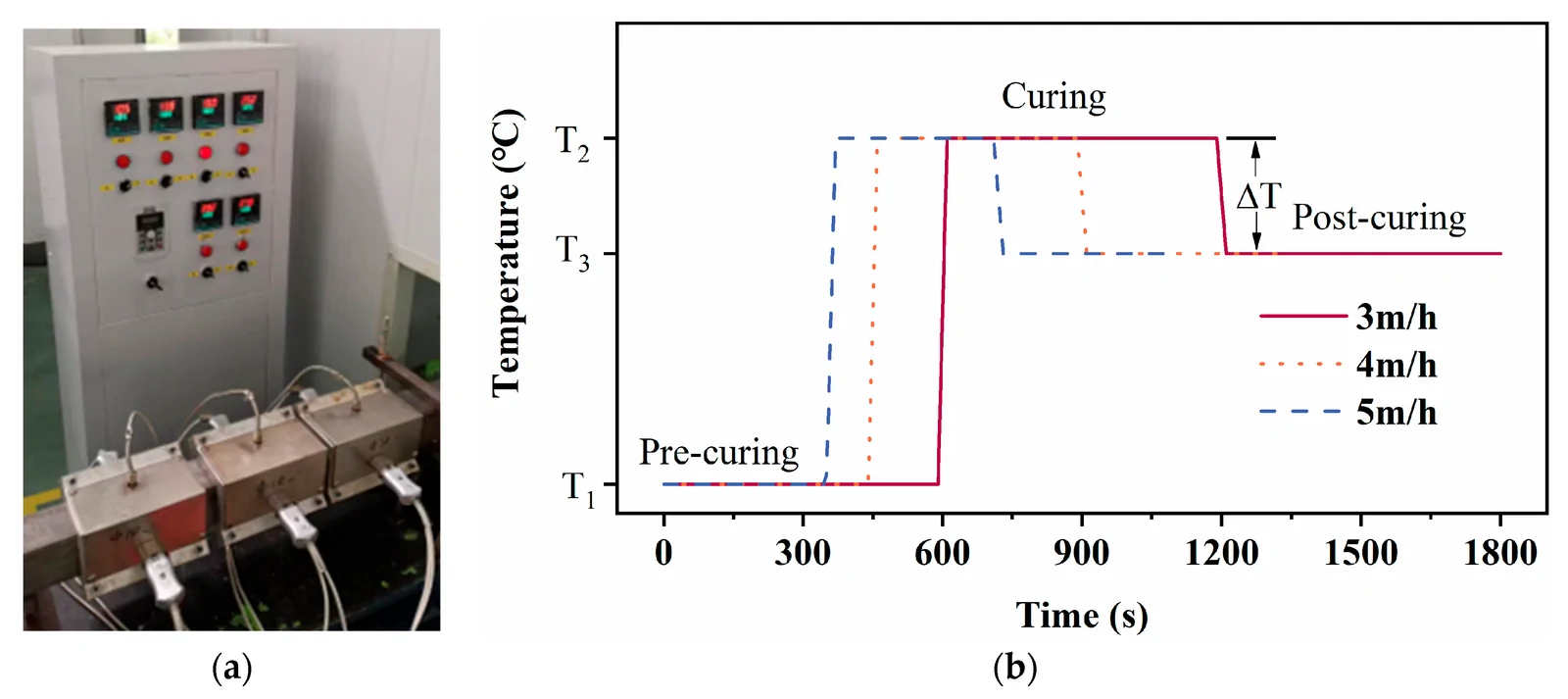
Curing process: (**a**) heated mold; (**b**) temperature curve of the inner surface of the mold.

During the production process of the core rod, when the production line reaches a stable state, by measuring the weight of the fibers per meter and the volume of the core rod, the volume of the fibers per meter is calculated based on the fiber density. The 10 measurement results are converted and averaged to obtain a fiber volume ratio of approximately 70%. The pultrusion speed is adjusted within the range of 3 m/h to 6 m/h. The mold adopts three-stage temperature zone heating, with each heating zone having a length of approximately 0.5 m, as depicted in Figure 7a; accordingly, the temperature curve on the inner surface of the mold is shown in Figure 7b. The curing process involves three stages, namely pre-curing, curing, and post-curing. The involved process parameters include the pultrusion speed, temperature settings T_1_ and T_2_ for the pre-curing and curing stages, and the temperature difference ΔT between the pre-curing and post-curing stages.

**Figure 8:**
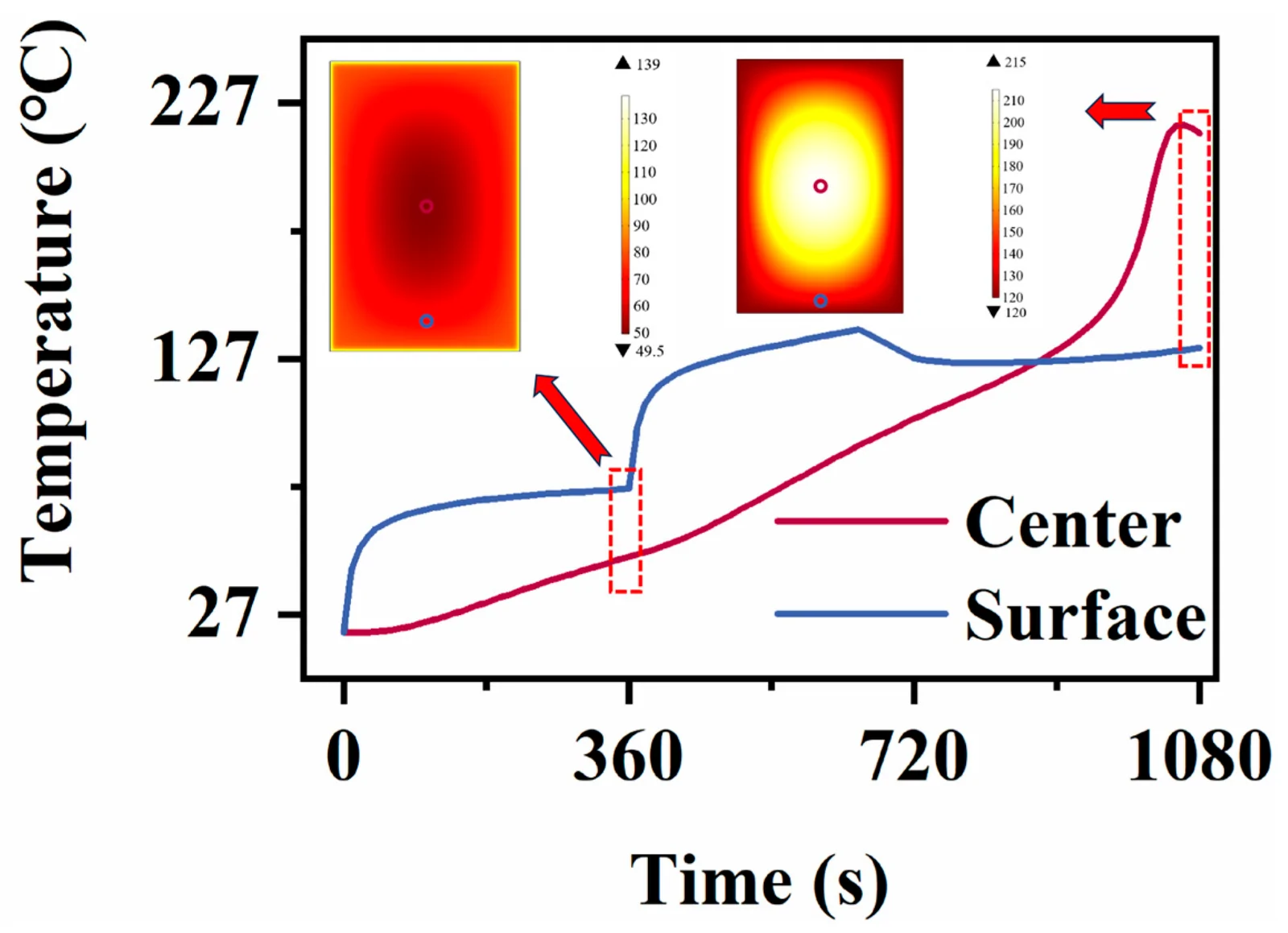
Internal temperature variation of the mandrel.

Taking a pultrusion speed v of 5 m/h, a pre-curing temperature T_1_ of 80 °C, a curing temperature T_2_ of 140 °C, and a curing temperature difference ΔT of 20 °C as an example, Figure 8 depicts the temperature evolution. The surface temperature is predominantly governed by conductive heat transfer from the heated mold wall and thus closely tracks the prescribed wall temperature profile. In contrast, the core temperature evolves under the competing effects of inward conductive heat flux from the surface and volumetric heat generation due to the exothermic reaction. During the early stage, heat conduction alone drives the core temperature rise, resulting in a pronounced thermal lag relative to the surface. However, once the reaction rate surpasses a critical threshold—reached at approximately 720 s—the accumulated exothermic energy within the core triggers a sharp temperature increase. Consequently, the core temperature overtakes the surface temperature and attains a distinct maximum peak.

**Figure 9:**
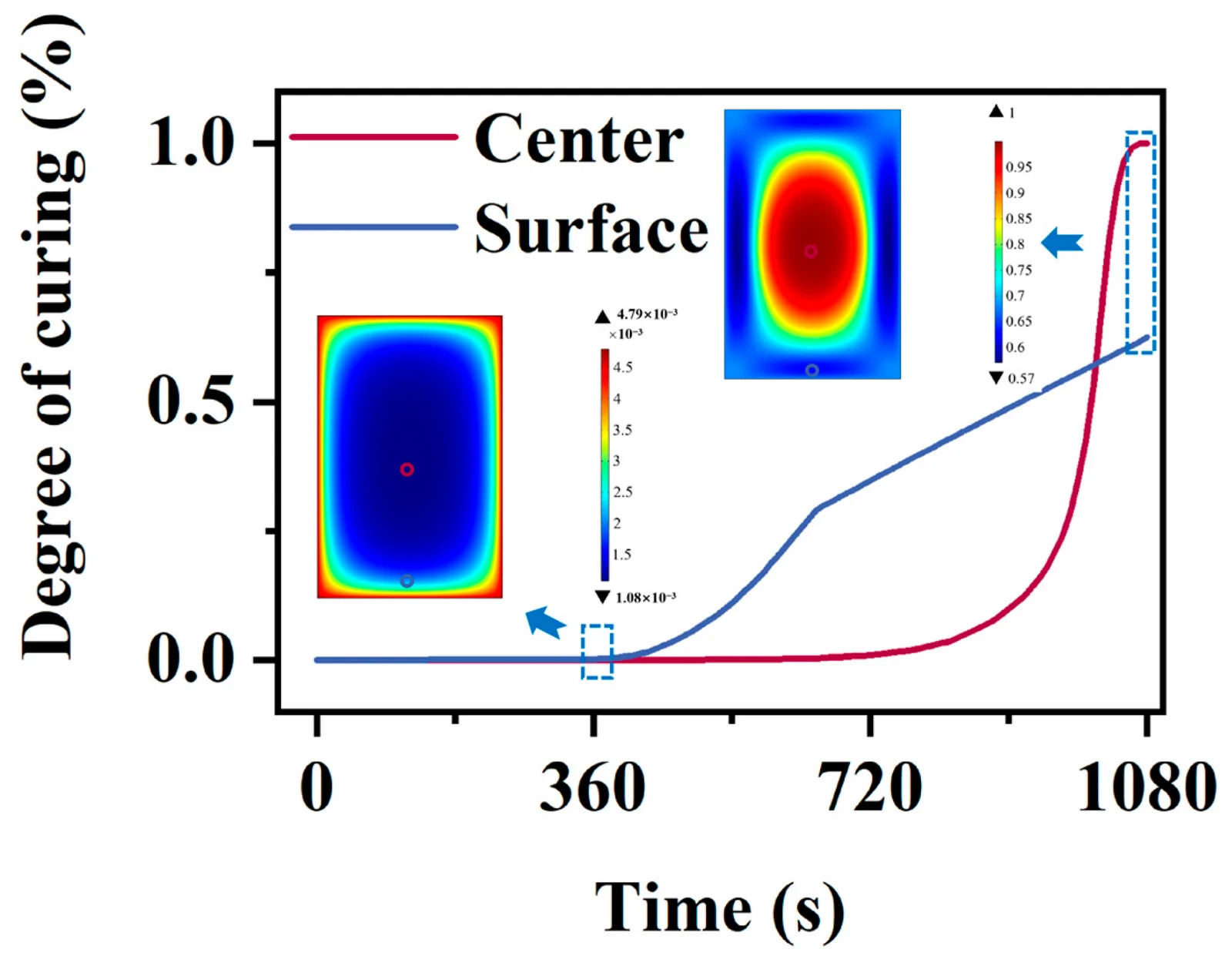
Change in internal curing degree of the mandrel.

Figure 9 depicts the evolution of the degree of cure at the core and surface regions. Similarly, the surface region initiates curing earlier because it reaches the reaction onset temperature (approximately 130 °C) sooner due to direct heating from the mold wall. Consequently, the surface curing degree initially outpaces that of the core. However, once the exothermic reaction is activated in the core, the accumulated heat accelerates the reaction rate, and the low thermal conductivity of the composite impedes heat dissipation, enabling the core to rapidly ‘catch up’ and eventually surpass the surface curing degree at approximately 900 s.

Thus, the final degree of cure at the surface, the degree of cure after the pre-curing stage, and the maximum temperature at the core region can be defined as the key curing characteristic parameters.

### 3.3. Analysis of the Influence of Process Parameters on Key Characteristic Quantities

An orthogonal experiment was employed instead of a full factorial experiment to achieve optimization of the core rod process. The experiment was designed in accordance with the L_9_(3^4^) orthogonal array, with factor levels detailed in Table 5. After inputting the parameters as specified in the orthogonal experimental table, the key curing characteristic parameters derived from simulations are presented in Table 6.

To quantitatively evaluate the influence of each process parameter on the curing characteristics, a range analysis was performed on the simulation results. For a given factor column *j* in the L_9_ array, the average value of the target characteristic *K_ij_* for each level i (i = 1, 2, 3) is calculated as Equation (13).

(13) $$K_{ij}=\frac{1}{3}\sum_{k=1}^{3}y_{ijk}$$

where *y_ijk_* is the simulation result corresponding to level *i* of factor *j* in the *k*-th experimental run. The range *R_j_* for factor *j* is then determined by Equation (14).

(14) $$R_{j}=max\left(K_{1j},K_{2j},K_{3j}\right)-min\left(K_{1j},K_{2j},K_{3j}\right)$$

The range Rj reflects the magnitude of the main effect of factor j on the target characteristic. Based on the calculated ranges, the four process parameters (pultrusion speed v, pre-curing temperature T_1_, curing temperature T_2_, and temperature gradient ΔT) can be ranked in descending order of their influence on each curing characteristic. The resulting range values and factor rankings are presented in Table 7.

The results of the range analysis for the orthogonal experiment are presented in Table 7. For the final surface degree of cure, the influences of curing temperature T_2_, pull-out speed v, and curing temperature difference ΔT are significantly stronger than that of pre-curing temperature T_1_. For the pre-curing degree, the effects of pre-curing temperature T_1_ and pull-out speed v are relatively prominent. For the maximum temperature, the order of influence magnitude is as follows: curing temperature T_2_ > pull-out speed v > curing temperature difference ΔT > pre-curing temperature T_1_.

### 3.4. Optimization of Process Parameters

Based on practical production experience, this study specifies that the final surface degree of cure should be in the range of 95% to 100%, the pre-curing temperature (T_1_) should be maintained below 3%, and the maximum temperature at the center should be minimized. The curing degree results listed in Table 6 were preprocessed using Equation (15), and the process characteristic data was normalized and scored according to Equation (16). The optimal process parameters are shown in Table 8.

(15) $$\left\{\begin{array}{c} \alpha_{1\;}^{*}={\;\alpha }_{1}-3\%\\ \alpha_{3}^{*}=\alpha_{3}-95\% \end{array}\right.$$

(16) $$S=\frac{\alpha_{1}^{*}-\alpha_{1-min}^{*}}{\alpha_{1-max}^{*}-\alpha_{1-min}^{*}}+\frac{\alpha_{3}^{*}-\alpha_{3-min}^{*}}{\alpha_{3-max}^{*}-\alpha_{3-min}^{*}}+\frac{T-T_{max}}{T_{max}-T_{min}}$$

## 4. Discussion

### 4.1. Analysis of Defect Causes

Based on the analysis of the thermal-curing degree field within the core rod during the curing process, it can be confirmed that the defects generated during core rod production are correlated with the variation in curing degree throughout the pultrusion process. Therefore, in this study, the production parameters of defective products were input into the model, and the formation mechanisms of defects in electric composite core rods were analyzed using curing degree variation curves.

**Figure 10:**
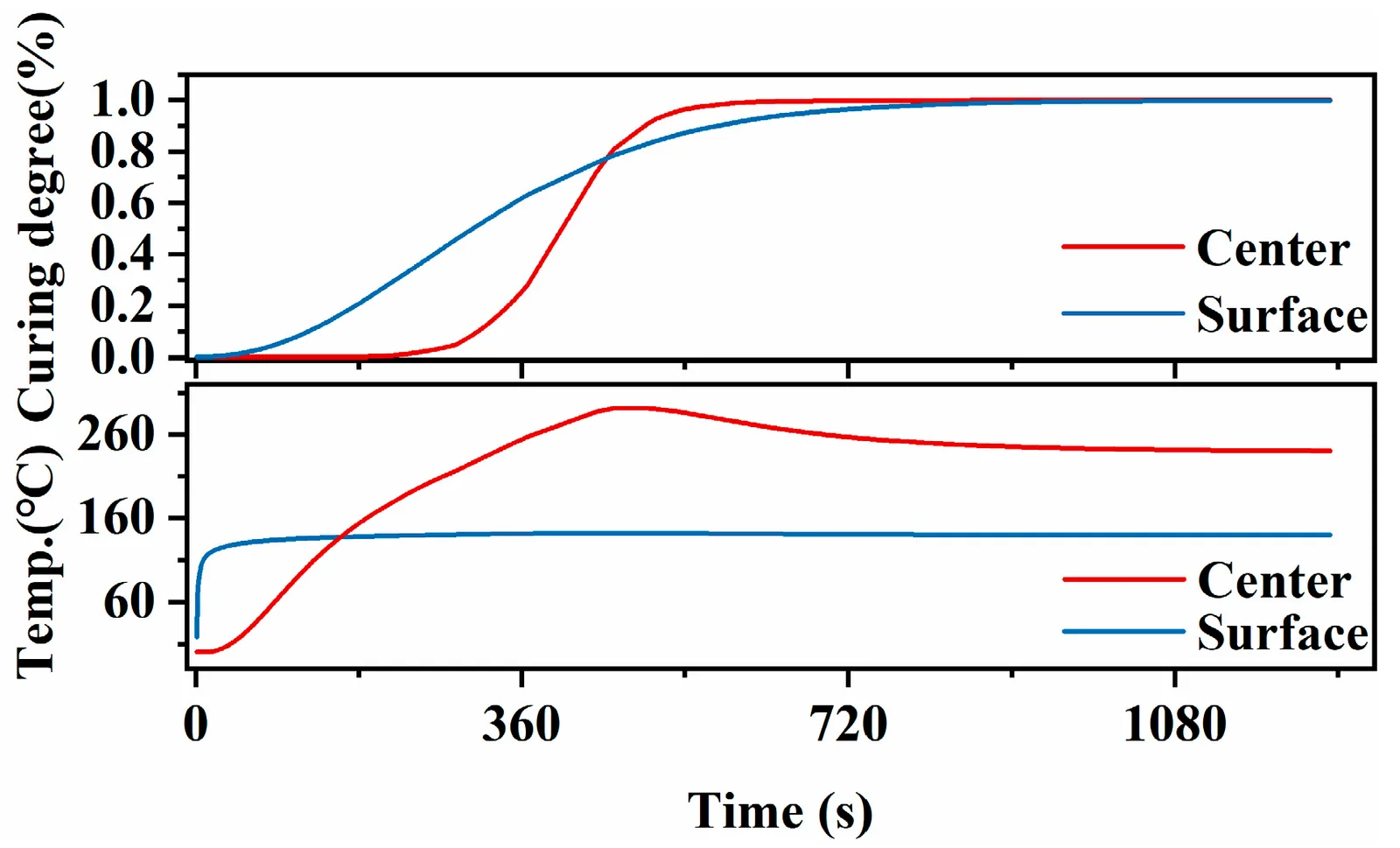
Thermal curing characteristic curve with internal pore defects.

**Figure 11:**
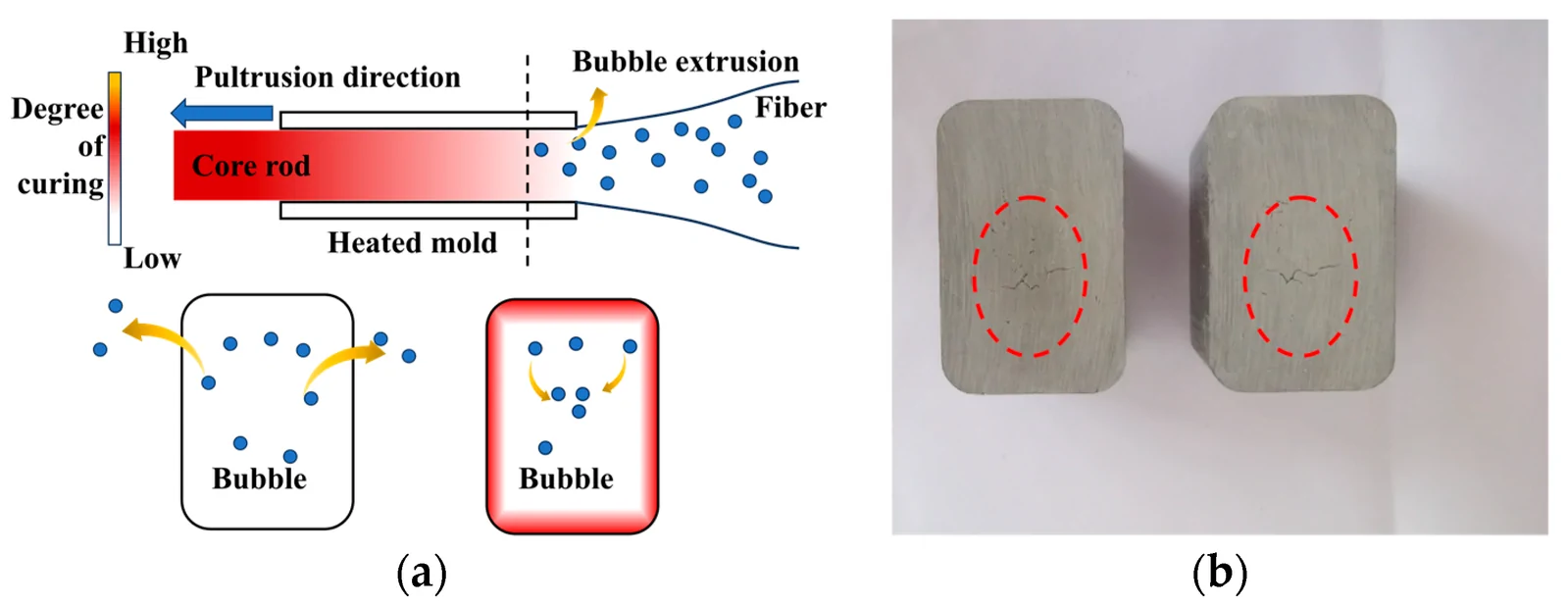
Causes of defects: (**a**) cause of internal pore defects in mandrel; (**b**) actual specimen.

Internal cracking occurred under the process parameters of pre-curing temperature 140 °C, curing temperature 140 °C, post-curing temperature 140 °C, pull-out speed 5 m/s, and fiber content 70%. The curing degree curve of the core rod is presented in Figure 10. Under these parameter settings, the curing degree of the surface layer exceeded 60% as early as the pre-curing stage (360 s), which induced the formation of internal cracks. As shown in Figure 11a, the excessively high temperature at the mold inlet accelerated the resin curing rate. This led to an increase in resin viscosity or the formation of an outer-surface self-skinning layer, which prevented the timely extrusion of bubbles in the resin mixture. During the core rod molding process, these bubbles were continuously compressed toward the center, eventually forming large pore defects at the core of the rod, as depicted in Figure 11b.

**Figure 12:**
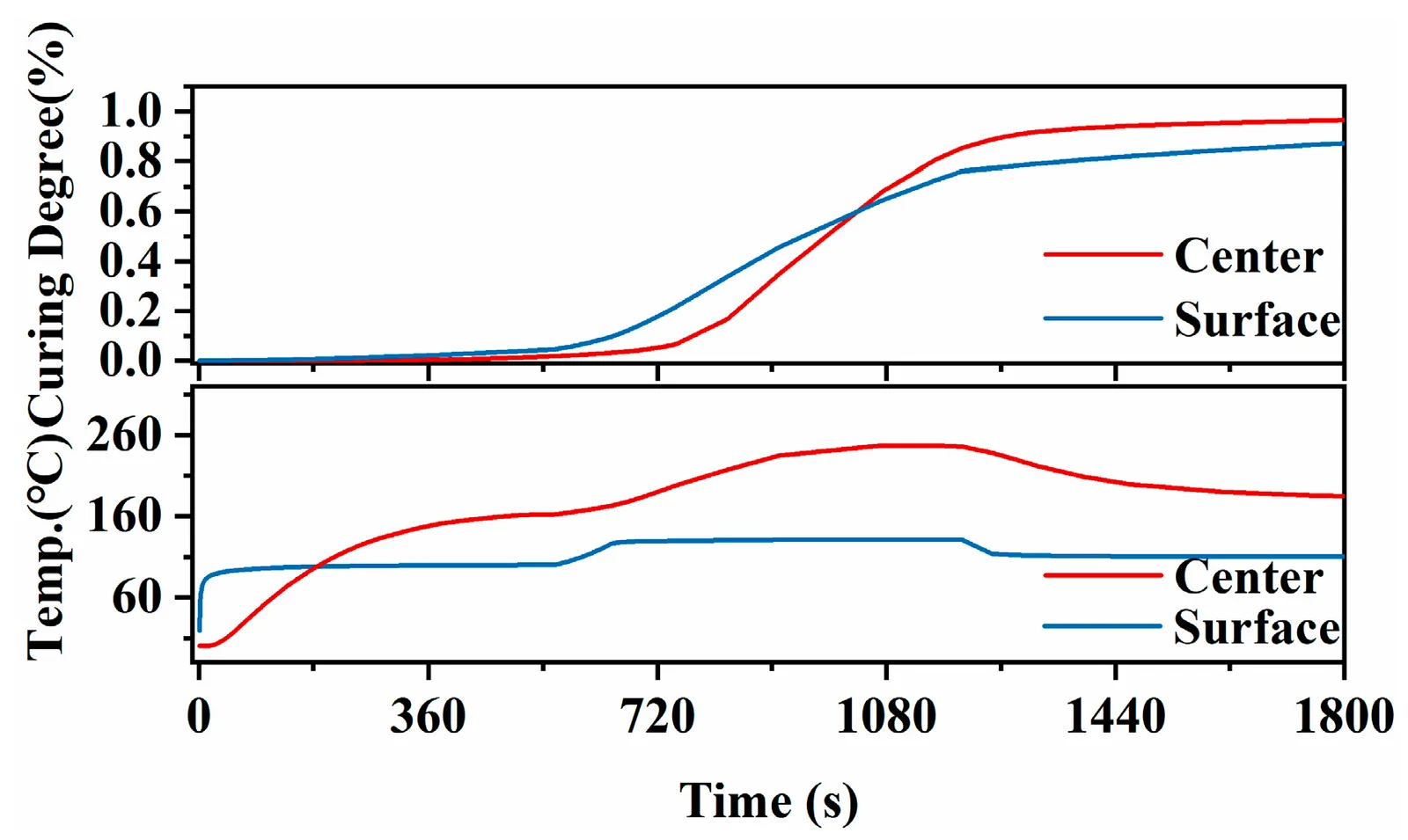
Thermal curing characteristic curve when surface cracks occur.

**Figure 13:**
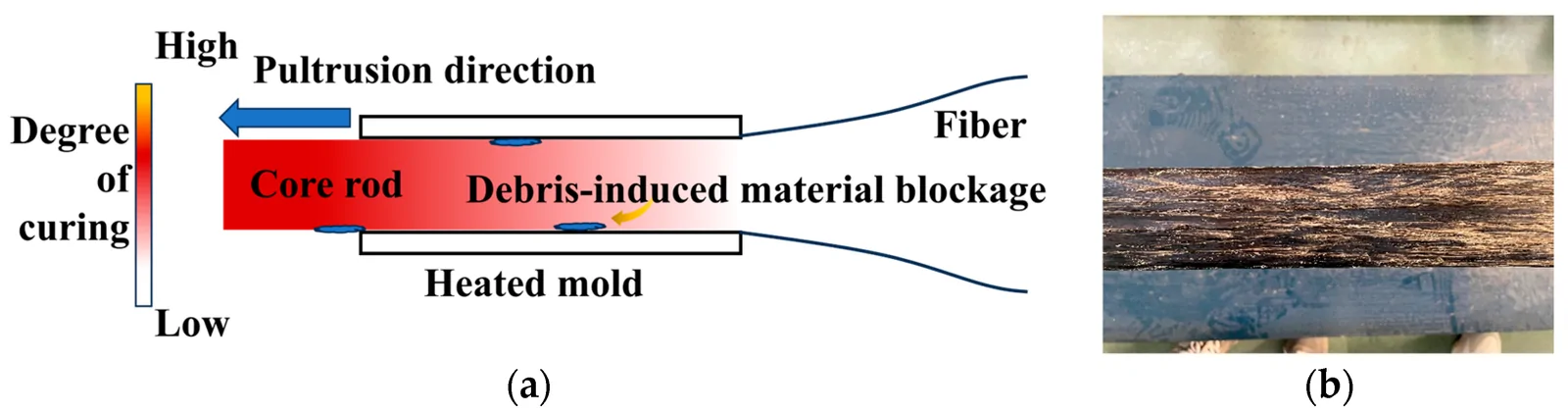
Causes of defects: (**a**) core rod surface crack defect cause; (**b**) actual image.

Surface cracking was observed under the process parameters: pre-curing temperature 100 °C, curing temperature 130 °C, post-curing temperature 110 °C, pull-out speed 3 m/s, and fiber content 70%. As presented in Figure 12, the temperature and curing degree curves under these parameters exhibit a trend consistent with the normal curing profile; thus, the primary contributing factor to the defect is deemed to be an excessively low pultrusion speed. As depicted in Figure 13b, resin debris and crack-like scratches are visible on the outer surface of the core rod. The underlying mechanism for this phenomenon is illustrated in Figure 13a: due to the low pultrusion speed and insufficient fiber content, the resin material that had cured and adhered to the inner wall of the mold failed to be entrained in a timely manner by the fiber pull-out process, leading to severe material blockage inside the mold. This accumulated material subsequently abraded and scratched the surface of the core rod passing through the mold.

**Figure 14:**
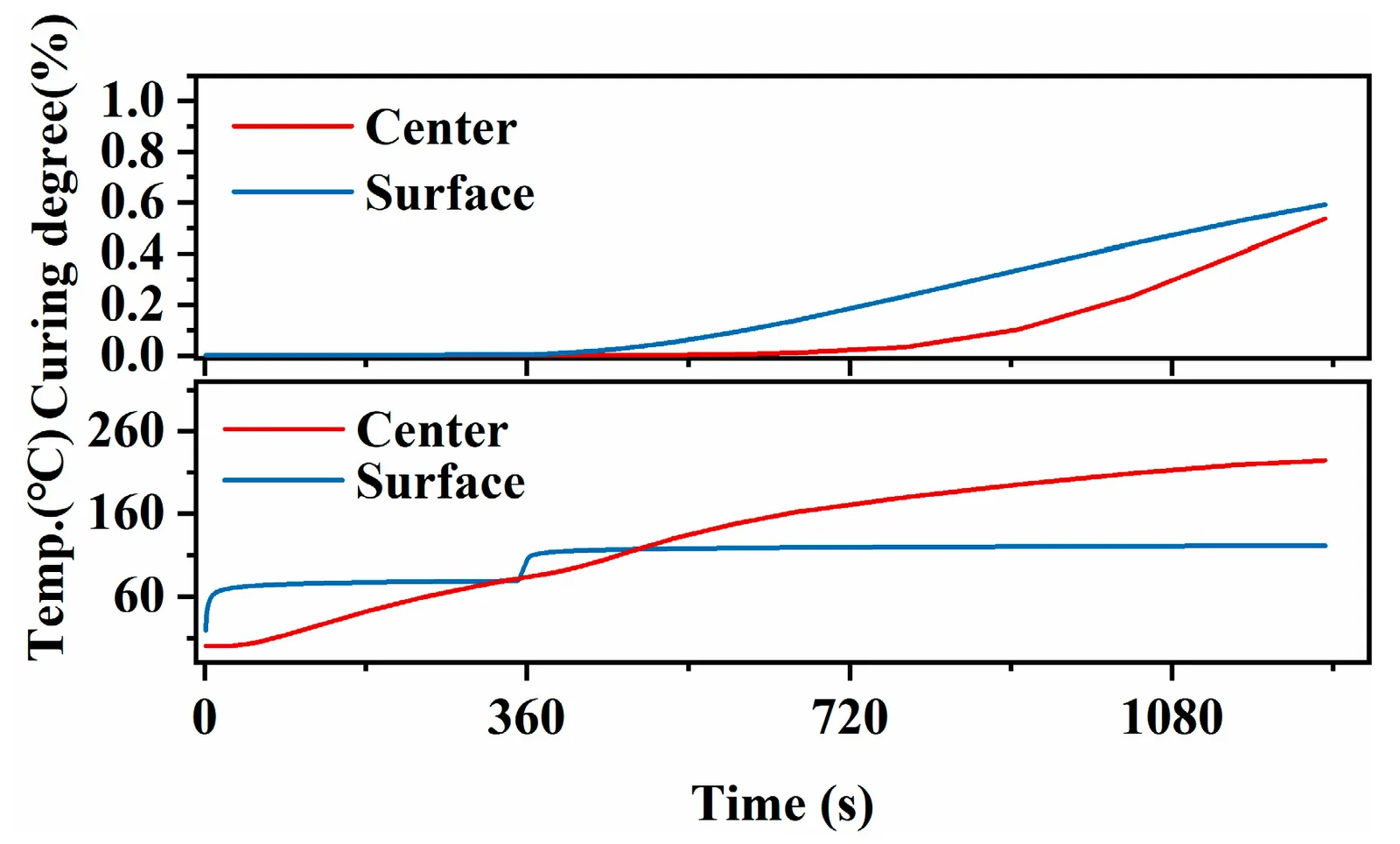
Thermal curing characteristic curve under insufficient curing.

**Figure 15:**
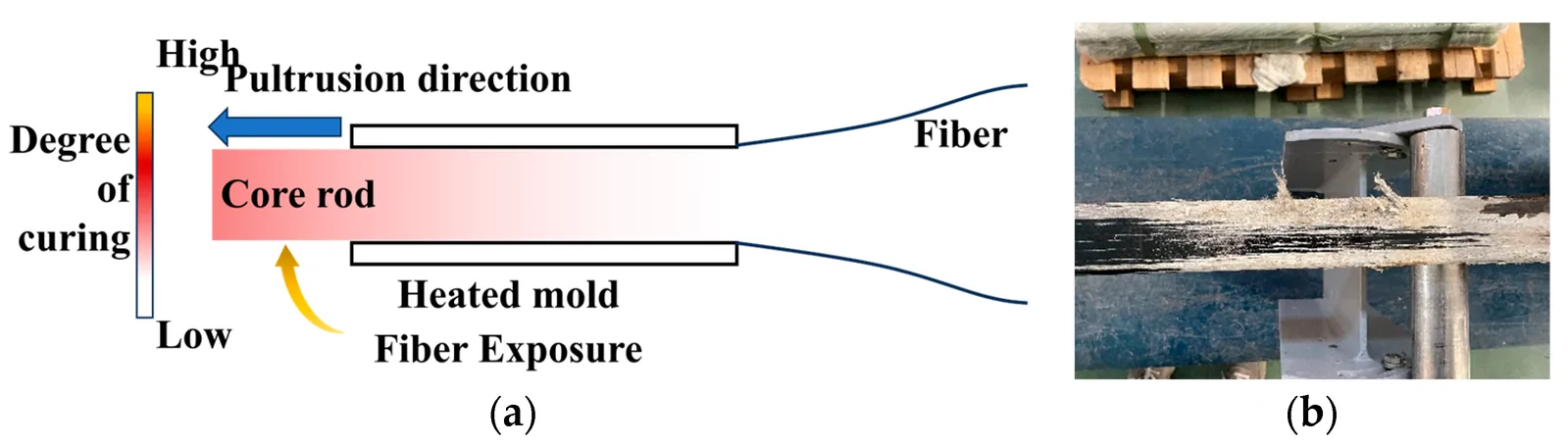
Causes of defects: (**a**) core rod insufficient curing defect cause (**b**) actual image.

Inadequate curing was observed under the process parameters: pre-curing temperature 80 °C, curing temperature 120 °C, post-curing temperature 120 °C, pull-out speed 5 m/s, and fiber content 55%. As presented in Figure 14, due to the combined effects of temperature and speed settings, the curing degree of both the core and surface layers failed to exceed 70%. Notably, excessive pultrusion speed or insufficient curing temperature will lead to significant under-curing of the core rod, as illustrated in Figure 15a. The specific morphological characteristics are shown in Figure 15b: the outer surface of the core rod lacks self-skinning, and fibers are exposed, which are typical visual defects associated with under-curing.

**Figure 16:**
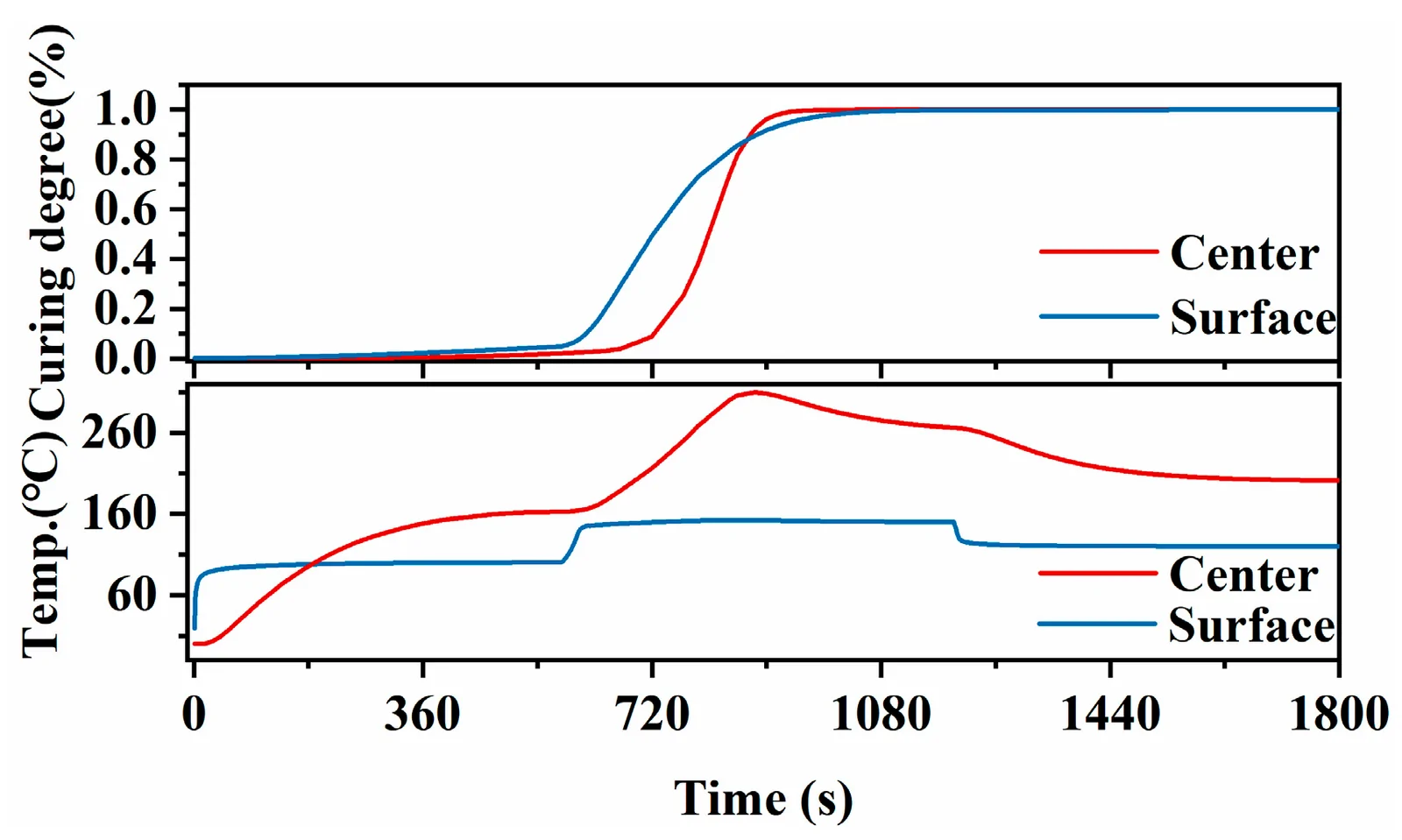
Thermal curing characteristic curve during over-curing defects.

**Figure 17:**
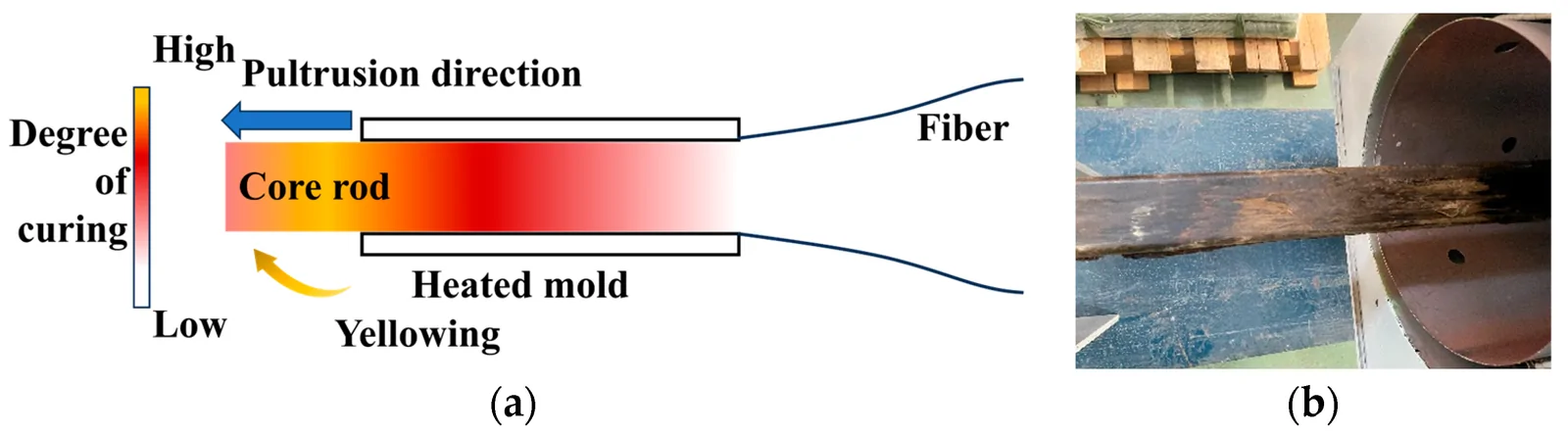
Causes of defects: (**a**) core rod over-curing defects cause; (**b**) actual image.

Resin yellowing was observed under the process parameters: pre-curing temperature 100 °C, curing temperature 150 °C, post-curing temperature 120 °C, pull-out speed 3 m/s, and fiber content 70%. As presented in Figure 16, under these parameter settings, the curing degree of both the core and surface layers approached 100% during the curing stage, while the peak core temperature exceeded 300 °C. As illustrated in Figure 17, excessive mold temperature leads to significant over-curing of the core rod. Specifically, the resin matrix inside the core rod completes curing prior to demolding; subsequent sustained over-curing within the mold induces high-temperature aging characteristics, which not only render the core rod brittle but also cause noticeable surface yellowing—a typical visual indicator of resin thermal degradation.

### 4.2. Performance Comparison Between Core Rods and Defective Core Rods Under the Optimal Process

To quantitatively evaluate the practical impact of the four types of defects identified in this study, a comparative experimental investigation was conducted on core rods fabricated under the optimal process parameters and those produced with deliberately induced defect conditions. The experimental results are presented in Table 9.

The bending strength of core rods fabricated under the optimal process was 727.97 MPa. Among defective specimens, those exhibiting surface fiber exposure, indicative of under-curing, showed the greatest reduction in bending strength (626.09 MPa), representing a 14.0% decline relative to the optimal condition. This pronounced degradation stems from insufficient crosslinking density, which directly undermines the composite’s load-bearing capacity. Specimens with internal cracks exhibited a moderate 6.7% reduction (679.14 MPa), as internal voids diminish the effective load-bearing cross-sectional area and intensify stress concentration under flexural loading. In contrast, specimens with surface cracks displayed only a marginal 2.2% reduction (712.32 MPa), since such cracks are localized near the surface and exert negligible influence on overall flexural rigidity. Notably, yellowed specimens retained a bending strength of 706.38 MPa—demonstrating that discoloration has no substantial impact on short-term mechanical integrity.

In sharp contrast, insulation performance revealed a markedly more sensitive response to defects. Specimens produced under the optimal process passed the test with a leakage current of 48 μA, well below the 100 μA acceptance limit. Likewise, yellowed specimens also passed, registering a leakage current of 46 μA, further confirming that discoloration, while visually apparent, does not impair dielectric barrier functionality under the tested conditions.

Conversely, all three structural defect types (internal cracks, surface cracks, and surface fiber exposure) failed the water diffusion test after 100 h of boiling. This far surpasses the 100 μA pass/fail threshold and indicates complete loss of insulation functionality—not merely marginal deterioration. These failures arise because structural discontinuities or incomplete curing introduce continuous pathways for moisture ingress and ionic conduction, thereby catastrophically compromising the dielectric integrity of the core rod.

## 5. Conclusions

This study established a thermal curing degree simulation model for core rod forming based on an autocatalytic curing model, systematically analyzed the thermal curing characteristics during basalt fiber core rod manufacturing, identified key curing characteristic parameters, evaluated the influence of process parameters on these critical indicators via orthogonal experiments, and investigated the root causes of defects in basalt fiber core rod production. The main conclusions are summarized as follows:

(1) Analysis of thermal curing degree characteristics during core rod curing revealed that the surface temperature of the core rod is positively correlated with mold temperature, while the internal temperature is governed by both heat conduction from the surface and internal exothermic heat sources. In the middle-to-late curing stage, due to cumulative heat release, the internal temperature exceeds the surface temperature and reaches a peak. Curing degree is linearly proportional to cumulative heat; the surface layer cures first, and the core curing degree subsequently surpasses that of the surface layer.
(2) Root cause analysis of curing defects indicated that excessively high pre-curing temperature or overly slow pull-out speed leads to over-pre-curing, resulting in internal crack defects; excessively high pre-curing temperature or overly fast pull-out speed causes over-pre-curing with insufficient internal curing, leading to surface crack defects; inappropriate pull-out speed or curing temperature results in either insufficient surface curing or premature full curing, inducing under-curing or yellowing defects.
(3) The optimal process parameters were identified as: pultrusion speed of 4 m/h, pre-curing temperature of 90 °C, curing temperature of 140 °C, and temperature gradient of 20 °C. Core rods produced under these parameters exhibit a bending strength of 727.97 MPa and a leakage current of 48 μA, successfully passing the water diffusion test. Internal crack, surface crack, and surface fiber exposure defects cause leakage currents to exceed 1000 μA and lead to test failure, despite retaining over 85% of the optimal bending strength. In contrast, the yellowing does not affect the dielectric properties but cannot pass the appearance inspection. These results confirm that the optimized parameters can effectively suppress the defects that seriously affect the insulation performance.

While the present model accurately captures the thermal–chemical coupling during BFRP core rod pultrusion curing, it is still necessary to introduce research on the curing stress field and make the model more in line with the actual production line.

From the perspective of polymer science, this study contributes to the fundamental understanding of curing behavior in thick-section thermosetting composites. From an engineering and industrial perspective, the validated simulation framework offers a practical tool for predictive process optimization, eliminating the need for costly trial-and-error adjustments.

## Figures and Tables

**Table 1:** Characteristic parameters of the curing exothermic process.

Heating Rate (°C·min^−1^)	Initial Temperature (°C)	Peak Temperature (°C)	Final Temperature (°C)	Total Reaction Heat (J/g)
5	132.1	153.3	175.2	125.1
10	142.0	164.5	184.7	106.25
15	151.0	173.5	192.2	108.62
20	156.0	179.3	197.7	108.85

**Table 2:** Curing reaction heat model parameters.

B (°C·min^−1^)	A	m	n
5	4.49 × 10^7^	0.3979	1.147
10	2.86 × 10^7^	0.3976	1.006
15	2.75 × 10^7^	0.4002	0.9422
20	2.08 × 10^7^	0.3586	0.7639

**Table 3:** Material parameters.

Material	Density (kg/m^3^)	Specific Heat Capacity (J/kgK)	Thermal Conductivity (w/mK)
Resin	1.24	1300	0.67
Basalt fiber	2.73	800	0.035

**Table 4:** Calculation error analysis.

**Number**	**v (m/h)**	**T_1_ (°C)**	**T_2_ (°C)**	**ΔT (°C)**	**Calculation of Curing Degree (%)**	**Actual Curing Degree (%)**	**Error (%)**
1	5	80	140	100	46.4	54.2	7.8
2	4	100	130	80	97.9	100	2.1
3	3	100	120	100	42.9	51.4	8.5

**Table 5:** Orthogonal experiment factors.

Level	v (m/h)	T_1_ (°C)	T_2_ (°C)	ΔT (°C)
1	3	80	130	0
2	4	90	140	20
3	5	100	150	40

**Table 6:** Orthogonal experiment results.

Number	v (m/h)	T_1_ (°C)	T_2_ (°C)	ΔT (°C)	Degree of Cure (%)	Pre-Cure Degree (%)	Maximum Temperature (°C)
1	3	80	130	0	83	0.89	226.8
2	3	90	150	20	97	2.10	272.6
3	3	100	140	40	84	6.01	249.7
4	4	80	150	40	90	1.25	273.5
5	4	90	140	0	92	2.29	252.2
6	4	100	130	20	58	4.01	211.2
7	5	80	140	20	57	0.48	218.5
8	5	90	130	40	40	1.16	111.5
9	5	100	150	0	96	3.30	272.3

**Table 7:** Results of range analysis.

Characteristics	v	T_1_	T_2_	ΔT
Surface curing degree	23.67	3	34	19.67
Pre-curing degree	1.35	2.59	0.91	0.65
Maximum temperature	48.93	32.3	89.63	38.87

**Table 8:** Optimal process parameters.

**v**	**T_1_**	**T_2_**	**ΔT**
5	80	150	0

**Table 9:** Comparison of mechanical and insulation performance between optimal and defective core rods.

Sample Type	Bending Strength (MPa)	Leakage Current (μA)	Water Diffusion Test Result
Optimal process	727.97	48	Pass
Internal crack	679.14	>1000	Fail
Surface crack	712.32	>1000	Fail
Surface fiber exposure	626.09	>1000	Fail
Yellowing	706.38	46	Pass

## Data Availability

The data presented in this study are available on request from the first authors and corresponding author.

## Abbreviations

The following abbreviations are used in this manuscript:

FRCMs	Fiber-reinforced composite materials
BFRP	Basalt fiber-reinforced polymer
DGEBA	Bisphenol A epoxy resin
MHHPA	Methylhexahydrophthalic anhydride
DMP-30	2,4,6-tris(dimethylaminomethyl)phenol
PDMS	Polydimethylsiloxane
DSC	Differential scanning calorimeter
